# Are sexually transmitted infections associated with male infertility? A systematic review and in-depth evaluation of the evidence and mechanisms of action of 11 pathogens

**DOI:** 10.1080/2090598X.2023.2218566

**Published:** 2023-07-05

**Authors:** Kareim Khalafalla, Walid El Ansari, Pallav Sengupta, Ahmad Majzoub, Haitham Elbardisi, Onder Canguven, Kareem El-Ansari, Mohamed Arafa

**Affiliations:** a Department of Urology, Hamad General Hospital, Doha, Qatar; b Department of Urology, University of Texas Health Science Center, Houston, Texas, USA; c Department of Urology, University of Texas MD Anderson Cancer Center, Houston, Texas, USA; d Department of Surgery, Hamad Medical Corporation, Doha, Qatar; e College of Medicine, Qatar University, Doha, Qatar; f Department of Population Health, Weill Cornell Medicine-Qatar, Doha, Qatar; g Physiology Unit, Department of Biomedical Sciences, School of Medicine, Gulf Medical University, Ajman, UAE; h Faculty of Medicine, Ain Shams University, Cairo, Egypt; i Department of Urology, Weill Cornell Medicine-Qatar, Doha, Qatar; j Department of Andrology, Cairo University, Cairo, Egypt

**Keywords:** Male infertility, semen quality, sexually transmitted infections

## Abstract

**Purpose:**

To systematically review the evidence on the association between sexually transmitted infections (STIs) and male infertility. We sought to answer two questions: Are STIs significantly associated with detrimental changes in semen parameters?; and, is the prevalence of STIs significantly higher in infertile than fertile men?

**Materials and methods:**

PubMed, Scopus and Google Scholar databases were searched (inceptionMarch 2023) following the PRISMA guidelines. Identified original studies in English on the association between STIs and male infertility were included. Data was tabulated/described by pathogen, mechanisms of action, number of studies and their level of evidence.

**Results:**

Seventy out of 903 originally retrieved articles were included in this review. For the detrimental changes in semen parameters (first question), the evidence seems equivocal based on the nearly equal number of studies and similar levels of evidence. The only exception was for Ureaplasma, where the number of studies and levels of evidence supported an association with male infertility. Pertaining to a significantly higher prevalence of STI among infertile compared to fertile men (second question), evidence was insufficient to support/deny a significant association. The two exceptions were Ureaplasma and Mycoplasma, where the number of studies and evidence levels were in favour of an association with male infertility.

**Conclusions:**

Generally, the relationship between STIs and male infertility remains to be uncovered. Our appraisal of the overall state of this relationship shows that the evidence base leaves much to be desired. The exceptions are Ureaplasma and Mycoplasma, where the evidence convincingly suggests their associations with infertility in men.

## Introduction

Sexually transmitted infections (STIs) are common clinical conditions with major implications for the patient and healthcare system [1]. The World Health Organization’s (WHO) report on global STI surveillance in 2018 indicated a worldwide incidence of almost a million new cases per day [2]. More than 30 different organisms were identified (including bacteria, viruses and parasites), transmitted sexually via vaginal, anal and oral routes. The four most common etiological STIs, chlamydia, gonorrhea, syphilis and trichomoniasis cause about 376 million new infections each year [3].

Infertility, defined as the inability to conceive even after 12 months of unprotected regular intercourse, is estimated to affect 15% of couples globally (≈48.5 million couples), and males contribute to 50% of these cases [4]. Whilst the association between STIs and female reproduction has been thoroughly examined [5,6], controversy surrounds the relationship between STIs and male fertility potential. In 2000, the WHO reported the association of STIs with male infertility for the first time [7]. Consequently, the potential impact of STIs on male reproduction gained attention and several studies started to investigate this relationship [8,9].

Several mechanisms might explain the effect of STIs on male infertility [10,11]. The most common is the direct effect of the organism on semen quality, resulting from spermatozoa apoptosis [12,13]. The pathological implications of apoptosis have been linked to abnormal semen parameters due to the presence of high levels of apoptotic protein FAS (Fas cell surface death receptor) [14], Bclx, p53 and annexin V in the ejaculated spermatozoa [15]. In vitro and in vivo investigations have revealed that sperm cell apoptosis can be triggered by bacterial infections [16,17], due to the increased production of reactive oxygen species (ROS) [18] and inflammatory markers [19] resulting in oxidative stress. Sperm membranes, abundant with phospholipids, saturated and polyunsaturated fatty acids, render them susceptible to increased ROS-induced damage caused by STIs and the resultant leukocytospermia. This may lead to sperm DNA fragmentation (SDF) and alterations in sperm morphology, thus resulting in reduced vitality [20].

Antibiotic treatment for STIs has been reported as another contributor to the altered semen parameters [21,22]. Key steroidogenic enzymes [23] and reproductive hormones are competitively inhibited by antibiotic treatment [24,25]. Moreover, antibiotics can induce direct functional impairment to the spermatogenic cell, Sertoli cells as well as an anatomical disruption of blood testes barrier [22,26]. The presence of antisperm antibodies was previously suspected to be related to STIs; however, studies have shown no such correlation [27]. Antisperm antibodies in cases of STIs are believed to be due to epididymal obstruction resulting from epididymitis and scaring in untreated STIs [28]. Furthermore, infection of the male accessory glands (prostate, epididymis) through primary infections or extension from initial site can result in scarring, fibrosis, and disturbed seminal microenvironment which affects semen parameters [29,30].

The literature reveals gaps. A few systematic reviews examined the associations between single sexually transmitted organisms and male infertility e.g. chlamydia trachomatis [31,32], human papillomavirus (HPV) [33–37] or Neisseria gonorrhoeae [38]. To the best of knowledge, only two narrative reviews exist, and both are outdated [9,39]. Another two systematic reviews assessed the relationships between STI and men’s sexual function/fertility; one is about 8 years old [40]; and the second was conducted very early in 2021, including articles published until 2020, and had a limited yield of 36 articles [41]. In addition, the recent years have witnessed an increased gush of men’s health publications [42,43]. Collectively, these considerations acted as the driver for the current systematic review.

Therefore, the current systematic review aimed to bridge this knowledge gap and provide a fresh and comprehensive assessment of the association between STIs and male factor infertility. The specific objectives were to answer two related questions: ‘Are STIs significantly associated with unfavorable changes in semen parameters?’; and ‘Is the prevalence of STI significantly higher among infertile compared to fertile men?’

## Material and methods

### Protocol

This systematic review was undertaken in accordance with the preferred reporting items for systematic reviews and meta analysis (PRISMA) guidelines [44,45] with additional guidance from the Cochrane handbook of systematic reviews and meta analysis for interventions [46]. The protocol for this systematic review was not registered in PROSPERO.

### Information sources and study selection

The search was performed using PubMed, Scopus, Google Scholar electronic databases and reference lists of included studies for articles published up to 1 March 2023. Multiple databases were searched to limit bias as recommended by the Cochrane collaboration [46]. In line with others [47], the search strategies were constructed from combinations of medical subject headings (MeSH) and keywords, and further adjusted for the individual databases.

### Search strategy

We used the keywords ‘Sexually transmitted infections’, ‘Sexually transmitted diseases’, ‘STI’, ‘STIs’, ‘STD’, ‘STDs’, ‘men’, ‘males’, ‘fertile’, ‘fertility’, ‘infertile’, ‘infertility’, ‘reproduction’ [in Title/Abstract]. The medical subject headings (MeSH) terms used were sexually transmitted infections (All Fields) AND ‘men’ (MeSH Terms); sexually transmitted infections (All Fields) AND ‘males’ (MeSH Terms); sexually transmitted infections (All Fields) AND ‘fertile OR fertility’ (MeSH Terms); sexually transmitted infections (All Fields) AND ‘infertile OR infertility’ (MeSH Terms); sexually transmitted infections (All Fields) AND ‘reproduction OR reproductive’ (MeSH Terms). Additional searches included specific organisms e.g. Chlamydia trachomatis, Hepatitis B, Hepatitis C, Neisseria gonorrhoeae, Herpes genitalis, Human papilloma virus, Trichomonas vaginalis, Ureaplasma urealyticum, Mycoplasma hominis, Human Immunodeficiency virus, Treponema pallidum, Herpes simplex. We also conducted further searches using the reference lists of studies and review articles for a selection of relevant articles. The references of all included articles or relevant reviews were crosschecked.

### Data management

Results from each database were imported into Mendeley desktop and duplications were removed. Three authors (KK, PS, MA) independently screened the titles and abstracts of the remaining studies for eligibility. Studies for which eligibility could not be gauged premised on the abstract were retrieved in full for further assessment. Titles and abstracts were screened, and the full text of relevant articles was subsequently reviewed before inclusion. Data were then extracted, crosschecked and verified.

### Inclusion/exclusion criteria

The inclusion criteria were (1) original studies, (2) English language, (3) published from inception through 1 March 2023, (4) assessed ‘STIs’ and ‘male fertility/infertility,’ and, (5) patients of any age, gender, and ethnicity. Only studies containing original research and reports on possible association or a cause and effect relationship between STI and male infertility were included. Exclusion criteria includes studies based on gender (females), species (animals), and article types (review articles, case reports or Editorials/correspondence, commentaries), and studies that did not include the outcomes or comparisons were also excluded.

### Data collection process and data items

A Microsoft Excel 2013 data extraction sheet was used to facilitate data collection. Extracted information included study design, patient population (number, fertility status, comparison group/s, etc.), organism species (e.g. bacterial, viral, protozoa, etc.), pathogen name (chlamydia, trichomonas, HPV, HIV, etc.), specimen used (e.g. semen, urethral, blood, questionnaire, etc.), and semen parameters (semen volume, total sperm count, viability, spermatozoa motility, progressive motility, sperm penetration rates, sperm tail swelling rate, normal forms, abnormal forms, antisperm antibodies, percentage of spermatozoa with defective DNA condensation, teratozoospermia, etc.). Data was tabulated and described by pathogen, by mechanisms of action and by number of studies and level of evidence of the studies.

## Results

### Search results

**Figure 1.**
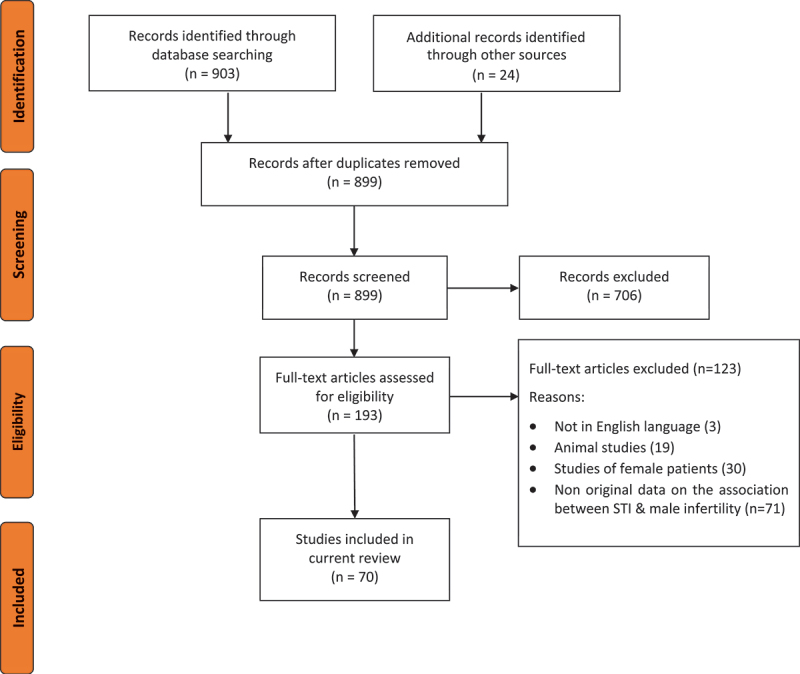
PRISMA flowchart of literature screening.

The literature search retrieved 903 articles, and an additional 24 records were identified from other sources. Duplicates were identified and removed (28 records). The remaining 899 articles were screened for suitability based on their titles and abstracts, and 706 nonrelevant articles were further excluded. The remaining 193 full text articles were assessed for eligibility of inclusion, and those without original research data on a possible association or cause and effect relationship between STI and male infertility (n = 71), as well as studies of animal species (n = 19) or female patients in the assessment of STI and infertility (n = 30), or not in English Language (n = 3) were excluded. The remaining 70 articles were included for analysis in the current review (Figure 1).

Table 1 shows the types of sexually transmitted organisms reported by the included studies, the numbers of studies reporting each, as well as the number of patients. The majority of studies were on Chlamydia trachomatis followed by Ureaplasma species and then Mycoplasma hominis. Viral infections were less studied; however, the most common virus represented in the included articles was human papilloma virus.

**Table 1.** Sexually transmitted organisms, numbers of reports and subjects.

Organism	Articles (n)^†^	Patients (n)
Bacteria		
Chlamydia Trachomatis	33	43,867
Ureaplasma	21	33,456
Mycoplasma Hominis	15	33,140
Neisseria Gonorrhea	5	2744
Virus		
Hepatitis B	4	6,014
Human Immunodeficiency	3	366
Cytomegalovirus	3	1,123
Herpes Simplex	4	1,265
Human Papilloma virus	6	952
Hepatitis C	2	70
Protozoa		
Trichomonas Vaginalis	3	1,139

^†^Some studies examined more than one organism

### Associations of STIs with male infertility

Table 2 outlines the individual studies addressing the effects of Chlamydia on male infertility. Of the total 26 studies on Chlamydia Trachomatis, 13 studies assessed the prevalence of the organism among fertile and infertile men. Of these, seven studies reported no effect of chlamydial infection on semen parameters, while five studies reported significant reduction of semen parameters with chlamydial infection. The source of samples used to test for the organism varied, where 20 studies used semen samples to test for the organism, while 11 studies used blood samples, 4 employed urethral smears, 3 used urine samples and one study utilized testicular tissue. Several studies used multiple sources.

**Table 2.** Studies on the associations of Chlamydia with male infertility.

Study Design	Patients/Specimen	Main results
Crosssectional [12]	293 infertile patients Semen	Chlamydia, mycoplasma and ureaplasma strains detected in 13%, 8.9% and 14% of men; Sperm DNA integrity not disturbed in men with semen contaminated by at least one of the investigated bacteria. % of spermatozoa with defective DNA condensation not higher in +ve semen samples vs ve ones
Crosssectional [48]	*CT* ve (n = 46) and +ve (n = 30) patients, healthy men (n = 53) undergoing vasectomy. Semen	No significant difference between *CT* +ve and ve patients in sperm count, progressive sperm motility, sperm morphology
Casecontrol [49]	127 IM,188 FM. Semen	No significant difference between *CT* +ve and ve patients in sperm count, progressive sperm motility, sperm morphology
Case-control [50]	93 IM, 70 FM. Semen, Urine	No significant difference in *CT* prevalence between IM & FM.
Case-control [51]	10 healthy volunteers; 1 *CT* positive patient. Semen	*CT* lipopolysaccharide is extremely toxic to spermatozoa → increased mortality rate
Case-control [52]	52 IM, 72 FM. Questionnaire, blood	CT prevalence not correlated with infertility at normal diagnostic antibodies titer >1:64. IM more likely seropositive for *CT* antibodies at titer 1:32 or less
Case-control [53]	90 IM, 190 FM. Blood	*CT* IgG (27.8 vs 6.3%) and IgA (22.2 vs 6.2%) elevated in men of infertile couples (*p* < 0.05)
Case-control [54]	181 IM, 367 FM Questionnaires + blood	Serological association between *CT* and subfertility; high incidence of asymptomatic infected IM
Case-control [55]	52 IM, 90 FM Questionnaires + blood	No significant difference in *CT* IgA or IgG antibodies titers between FM and IM
Case-control [56]	621 IM; 615 FM Semen	No significant difference in *CT* prevalence between IM & FM. No significant difference in semen parameters between *CT* +ve and -ve patients
Cross-sectional [57]	250 IM Semen, blood, urine	*CT* (PCR positive) had only lower semen volume compared with men without infection. Different prevalence of *CT* in IM according to specimen and test used; urine PCR 4.4%; Blood: IgM 1.2%, IgG 18.0%, IgA 0%; semen not detected; elevated IL-6/IL-8 in *CT* +ve men; IL-8 correlated with semen volume and patient’s age
Cross-sectional [58]	2607 infertile men Semen, Urine	No significant difference in all semen parameters between *CT* infected and not-infected groups
Cross-sectional [59]	454 men with *CT* prostatitis, 707 with other bacteria prostatitis Blood + semen	68.5% in *CT* group vs 1.9% in non- *CT* group were sub-fertile according to WHO criteria (p < 0.05)
Case-control [60]	40 symptomatic *CT* +ve cases; 20 controls. Semen	Sperm concentration, motility and morphology were significantly reduced in *CT* patients; strong correlation between mucosal anti–*CT* IgA and sperm concentration, sperm motility, and normal morphology
Case-control [61]	60 IM, 40 healthy controls. Urethral swabs from IM	No significant difference in *CT* prevalence between IM & FM
Case-control [62]	281 IM; 100 fertile controls. Semen	PCR detected *CT* in 13.9% of IM vs 2% of FM
Case-control [63]	135 IM, 88 FM Blood, semen, urethral smears	No significant difference in *CT* prevalence between IM & FM
Cross-sectional [64]	29 subjects. Semen	Incubation of elementary bodies of serovar E (Ct) caused significant decline in sperm motility (*p* < 0.05), increase of % of dead sperm (*p* < 0.05)
Case-control [65]	14 azoospermic men, 22 controls (post-vasectomy azoospermia) Testicular biopsy + surgical sperm retrieval	No presence of *CT*–specific DNA detected by PCR in epididymis/testis of 36 asymptomatic men with obstructive azoospermia; Unrecognized, asymptomatic *CT* infection does not lead to complete bilateral obstruction of male genital tract
Cross-sectional [66]	17,764 men. Semen	Infertility diagnosed in 1.27% of PCR polymerase chain reaction-positive and 1% of *CT*-negative men (no significant difference)
Cross-sectional (*CT, HPV*) [67]	1003 patients were enrolled. Semen, blood, urethral swabs, urine	HPV coinfection with *CT* had significantly lower % motile sperm and % normal morphological forms compared to *CT* infection alone; No differences of semen volume/ pH; No correlation between *HPV* genotypes, mucosal IgA type and semen variables
Cross-sectional [68]	627 healthy sperm donors. Semen	*CT* detected in 21.7%; significant difference between *CT* +ve and -ve patients in sperm motility and sperm morphology (lower in CT+ve)
Cross-sectional [69]	197 IM Semen + blood	No relationship between presence of seminal *CT* antibodies and sperm parameters, semen cultures, local ASA and sperm penetration testing as an indicator of functional capacity
Cross-sectional [70]	1303 sub fertile Semen + Blood	*CT* serology revealed 12.6% of men (166/1317) had *CT* Ab positive titres; No relationship of *CT* IgG Ab with male factor; No difference for semen parameters with regards to *CT* serology
Cross-sectional [71]	374 patients Semen + urethral swabs	Positive *CT* rate 2.92% in males; *CT* infection had no adverse effect on ART, regardless of whether infection present in one or both partners
Cross-sectional [72]	128 sub fertile Semen + and blood	No association of serum Ab to *CT* HSP60 with outcome of sperm analysis; No relationship with sperm count, % motility after liquefaction, morphology, including head abnormalities
Cross-sectional [73]	195 infertile men Semen	Prevalence of *CT* 3.6% in men with leukocytospermia; All semen parameters not significantly different between Leukocytospermia with STIs and without STIs
Cross-sectional [74]	7154 infertile men. Semen	5.8% prevalence of *CT* in IM; *CT* infection significantly correlated with abnormally low semen volume, increased white blood cell count and granulocyte elastase level; other routine semen parameters, antisperm antibody level and sperm acrosin activity not negatively impacted by *CT*
Case-control [75]	200 primary infertile men, 65 healthy controls. Semen	*CT* prevalence 14% in patients and 9.2% in controls. Significant associations between *CT* infection and asthenozoospermia, and abnormal vitality
Case-control [76]	153 infertile males, 74 healthy controls. Semen, urine, blood	*CT* prevalence 1.26% estimated from semen samples using PCR. Anti-*CT* antibodies IgM- 4%, IgA 28.16% and IgG-12.5% detected among IM vs 0% in fertile controls
Case-control [77]	5464 primary infertile men, 404 healthy controls. Semen	*CT* prevalence was significantly higher in patients than in control individuals (5.3% vs 2.0%)
Cross-sectional [78]	2415 infertile individuals. Semen	*CT* prevalence 48.61%; 170 CT+ samples received antibiotic. 78.82% of *CT*+ had significant reduction in % of infected spermatozoa after treatment; 59.7% decreased to non–detectable levels. Sperm morphology and motility significantly altered in *CT*+ pre–treatment vs controls. *CT* treatment significantly increased normal morphology, decreased mid–piece and tail defects and teratozoospermia index
Case-control [79]	50 infertile couples, 50 fertile couples. Semen	*CT* detected in 10% of semen samples of IM vs 0% in controls; sperm motility significantly lower in *CT* infected infertile patients than uninfected IM

-ve negative; +ve: positive; NG *Neisseria Gonorrhea*; MH *Mycoplasma Hominis*; UU *Ureaplasma Urealyticum*; DFI Sperm DNA fragmentation Index; *CT Chlamydia trachomatis*; NPPCR-nested primer polymerase chain reaction; ASA Antisperm Antibodies; IM infertile men; FM fertile men; ART Assisted reproductive techniques; PCR polymerase chain reaction

Table 3 outlines the individual studies discussing the effects of bacteria other than Chlamydia on male infertility. Of the total 20 studies, 11 assessed the prevalence of the organism among infertile and control men. Ten of these 20 studies reported the influence of these organisms on semen parameters and on fertility, while 4 found no significant effect. The source of samples used to test for the organism varied, but were mainly semen samples (17 studies), 3 employed urethral swab, 3 studies used urine samples and while 1 study used blood samples. Several studies used multiple sources.

**Table 3.** Studies on the associations of bacteria (other than Chlamydia) and with male infertility.

Study, Design (Organism)	Patients/ Specimen	Main Findings
Case-control *(NG)* [50]	93 IM, 70 FM. Semen, Urine	*NG* found in 6.5% of IM, 0 in FM (*p* < 0.05)
Case-control (*UU, MH*) [56]	621 IM; 615 FM Semen	No significant difference in *UU, MH* prevalence between IM & FM; *UU* was related, in infected compared with uninfected men, to lower mean sperm concentration and lower vitality; No significant difference in semen parameters between *MH* +ve and -ve patients.
Cross-Sectional (*MG, UU, NG*) [58]	2607 infertile men Semen, Urine	DFI was significantly higher in *UU* and *MG* infected men compared to non-infected; No significant difference in other semen; No significant difference in all semen parameters between *NG* infected and not-infected groups
Case-control (*MH, UU)* [62]	281 IM; 100 fertile controls. Semen	PCR detected *UU & MH* in 19.2% vs 11% & 9.9% vs 3% in IM vs FM, respectively
Case-control (*MH, UU)* [63]	135 IM, 88 FM Blood, semen, urethral smears	Significant difference in prevalence of *MH* between FM and IM; No difference in prevalence if considering *UU* alone
Cross-sectional (*UU, UP, MH, MG, NG*) [73]	195 infertile men. Semen	Prevalence of *UU, UP, MH, MG*, and *NG* were 8.7%, 21.0%, 8.2%, 2.1%, and 0%, respectively. All semen parameters not significantly different between LCS with STIs and without STIs, except semen volume in *MG*-infected patients with LCS was significantly lower than that in noninfected group
Case-control (*Usp, MH*) [77]	5464 primary infertile men, 404 healthy controls. Semen	Overall, prevalence of *Usp* and *MH* urogenital infection was significantly higher in patients than in control individuals (22.8% and 7.4% vs 17.8% and 1.7%, respectively)
Case-control (*MG, UP*) [79]	50 infertile couples, 50 fertile couples. Semen	*UP* detected in 12% of semen samples of IM vs 0% in controls; *MG* was not detected in any sample; sperm motility significantly lower in *UP* infected infertile patients than uninfected IM
Case-control (*UP, UU, MH*) [80]	Infertile males; 25 patients & 25 control group Semen + urine	Agent positivity was found in 12/25 patients in leucocytospermia group and 9 of 25 patients in control group; No significant difference was observed in prevalence. No significant negative effect of agent positivity on semen analysis parameters in either group
Case-control (*UU, MH*) [81]	19,098 infertile men; 3368 fertile control group. Semen, urethral specimens	Significant difference in *UU, MH and mixed pathogen* prevalence between IM & FM (10.22% vs 3.65%, 3.16% vs 0.89%, 1.8% vs 0.48 respectively); Significant differences in progressive/total motility, and normal forms between *UU*-infected, mixed infection, and uninfected groups (*p *< 0.05)
Cross-sectional (*MH*) [82]	3 healthy men. Semen	Sperm viability not altered by *MH* infection
Case control (TV) [83]	Healthy volunteers. Semen	Motility of sperm incubated with particulate fraction not much different from control
Cross-sectional (*TV*) [84]	8 volunteers Semen	Marked retardation in spermatozoa motility occurred when semen was mixed with concentrations of greater than 10” organisms per ml *TV*
Case control (*TV*) [85]	1131 asymptomatic men; 52 non infected. Semen	Increased seminal fluid viscosity and % particulate debris in infected group (*p* < 0.05); No difference in semen pH; Spermatozoa motility and morphologically normal forms decreased (*p* < 0.05); change in membrane integrity (*p* < 0.05) (hypoosmotic swelling test); significant improvement in semen characteristics in 25 cases after single course of treatment
Case-control (*UU, MH*) [86]	50 infertile; 48 fertile semen	Significant difference in *UU, MH* prevalence between IM (14% vs 6.3%, 48% vs 25% respectively)
Case-control (*UU*) [87]	31 infertile males with genital *UU*; 23 infertile males without; 27 normal volunteers. Semen	IM had significantly lower sperm volume, pH, density, percentage of forward, movement of sperm, sperm activate rate, sperm survival rate, and normal rate of sperm morphology than FM
Cross-sectional (*UU*) [88]	8 patients with positive infection (6 fertile/ 2 infertile). Semen	Sperm viability, motility, and morphology remained unchanged
Case control (*MH*) [89]	165 infertile males, 165 fertile men. Semen	Significant difference in *MH* prevalence between IM & FM; after antibiotic treatment, all semen parameters, except volume, pH, and viscosity, significantly improved, leukocytes in seminal fluid eliminated (*p* = 0.04), level of TAC elevated (*p* < 0.05), and ROS level as well as ROS/Total anti-oxidant capacity ratio reduced after antibiotic treatment (*p* < 0.05)
Cohort (NG) [90]	45 men with gonococcal epididymo-orchitis. Questionnaires, semen, blood	14/45 men had previously fathered children; Two years after gonococcal infection, 21% of fathers and 40% of whole group showed normal semen parameters; Bacterial gonadal infection may result in permanent azoospermia or oligospermia and may results in male related barrenness
Case-control *(UU* & *MH)* [91]	145 infertile, 49 fertile controls. Questionnaires, urine	No significant difference in *UU, MH* prevalence between IM & FM
Cross-sectional (*UU*) [92]	6 healthy donors. Semen, urethral smears	Significant decrease in % of active motility (*p* < 0.05) when *UU* added to washed spermatozoa; Significant affection of membrane permeability (hypo-osmotic swelling test) on spermatozoa; No differences in morphology characters between controls and experiments after addition of *UU*
Cross-sectional (*UU*) [93]	346 IM. Questionnaires, semen	*UU* found in 39.31%; *UU* infection associated with higher semen viscosity, lower semen pH, and reduced sperm concentration (all *p* < 0.05)
Case-control *(UU)* [94]	1461 IM, 375 FM. Semen	Significant difference in *UU* prevalence between IM & FM (38.77% vs 9.06%)
Case-control (*Usp*) [95]	540 infertile, 260 fertile. Semen	Significant difference in *Usp* prevalence between IM & FM (39.6% vs 19.2%)
Case-control *(UU)* [96]	100 infertile, 100 healthy controls. Semen	Significant difference in *UU* prevalence between IM & FM (12% vs 3%)
Case-control (*MG, NG, TV*) [97]	2000 infertile men, 248 normal fertile controls	*MG* prevalence 1.1% in patients, 0% in controls; *NG* and *TV* no detected in any patient, Higher seminal concentration of neutrophils and IL-6 among *MG* positives compared with STI negatives
Cross-sectional (*UU, UH*) [98]	212 IM, Semen	*UU* and *MH* infection detected in 17% and 23.6% of patients respectively, coinfection detected in 3.8%. *MH* infection and coinfection with *UU* associated with impairments in sperm quality

UP *Ureaplasma parvum*; MH *Mycoplasma Hominis*; UU *Ureaplasma urealyticum*; TV *Trichomonas vaginalis*; CT *Chlamydia Trachomatis*; DFI Sperm DNA fragmentation index; MG *Mycoplasma genitalium*; NG *Neisseria gonorrhoea*; Usp *Ureaplasma species*; TAC total anti-oxidant capacity; LCS leukocytospermia; IM infertile men; FM fertile men

Table 4 outlines the individual studies appraising associations of viruses with male infertility. Of the total 14 studies, 7 assessed prevalence of the organism among infertile men and controls. Ten of these 14 studies found deleterious effect of these viruses on semen parameters and fertility, while only 2 found no significant effect. The source of samples used to test for the organism varied, but were mainly semen samples (14 studies), and 3 studies additionally used blood samples.

**Table 4.** Studies on the associations of viruses with male infertility.

Study, Design (Organism)	Patients/ Specimen	Main Findings
Cross-sectional (HSV) [73]	195 infertile men. Semen	Prevalence of HSV was 2.1%. All semen parameters not significantly different between LCS with STIs and without STIs
Cross sectional (HIV) [99]	33 HIV seropositive men Semen + blood	Sperm vitality, motility, total motility, penetration rates significantly higher in patients with CD4+ counts >350/ml vs those with CD4+ counts <350/ml (P < 0.05); above parameters mentioned significantly correlated with CD4+ cell number (all P < 0.05); Significant differences in total sperm count and sperm tail swelling rate between patients co-infected with STI and without STI (P < 0.05)
Cross-sectional (HPV) [100]	100 men with intercourse history, 100 without. Semen	HPV associated with reduced sperm motility (53.7% in HPV-negative vs 37.7% in HPV-positive; p < 0.05); Other semen parameters did not differ by HPV status
Cross sectional (HSV) [101]	279 IM Semen	Total HSV-positive samples and HSV-2 positive samples associated with hematospermia (P = 0.03 each); More males with HSV infection and with HSV-1 infection had teratozoospermia than non-infected males (30% vs 23%, 38% vs 22.8%, respectively, NS). More males with HSV-2 infection had hypospermia and abnormal viscosity than non-infected males (22.2% vs 10%, 22.2% vs 13.3%, NS)
Cross-sectional (HPV) [102]	22 patients of partners of HPV +ve women with high-grade squamous intraepithelial lesion. Semen (Total/ semen fractions)	45% (10/22 patients) had the infection in total semen sample; HPV test positive in three samples also after swim up technique (semen fraction); HPV genotyping showed that all positive samples contained at least one high-risk genotype and there was prevalence of HPV16 and HPV18 (60%)
Case–control (HCV) [103]	57 HCV infected men; 40 fertile controls Semen	Semen volume, sperm count, sperm motility and abnormal morphology were significantly worse in *HCV* infected men compared to controls (2.33 vs 2.15 ml, 40.1 vs 75.4x10^6^/ml, 39.6% vs 58.1%, 40.35% vs 12.6%)
Case control (HBV) [104]	30 normal volunteers; 30 IM without HBV; 30 infertile males with HBV. Semen	Semen volume, pH, sperm density, % of sperm forward movement, sperm activation rate, survival rate, rate of normal sperm morphology significantly lower in IM *HBV* +ve than *HBV* -ve and FM; IL-17, IL-18, and MDA levels significantly higher in IM *HBV* +ve than *HBV* -ve and normal males
Case–control (HIV) [105]	250 HIV-seropositive men; 38 fertile controls. Semen	Semen volume, sperm concentration, sperm motility and median rapid and linear motility were significantly lower in *HIV* infected men compared to controls (1.8 vs 2.9 ml, 62 vs 100x10^6^/ml), 52% vs 64%, 14% vs 21%)
Case–control (HBV & HCV) [106]	15 men with chronic HBV; 3 men with chronic HCV; 20 fertile controls. Semen	No significant difference in sperm concentration, motility, or morphology between patients and controls Increased incidences of sperm necrosis in infected men (43.2% for HVC, 35.86% for HBV, and 15.57% for noninfected men (*p* < 0.05); Increased incidences of sperm apoptosis in infected men (6.76% for HVC, 7.5% for HBV, 2.9% for noninfected men; *p* < 0.05)
Cross-sectional (CMV) [107]	232 men attending infertility clinics. Semen	CMV more prevalent in IM with chronic inflammatory urogenital tract diseases compared with other groups combined (*p* < 0.05); CMV associated with reduced sperm count (39.5 vs 72.5 x 10^6^/ml, *p* < 0.05); HHV-6 more prevalent in fertile men with chronic urogenital tract inflammation than other groups combined (*p* < 0.05)
Case–control (HBV) [108]	5138 men with HBV, 25,690 noninfected controls. Semen	HBV infection associated with an increased 10-yr incidence of infertility diagnosis (p < 0.05)
Cross-sectional (HBV) [109]	831 infertile couples. Semen, blood	HBV infection detected in 61 male partners (7.3%); HBV infection incidence in male partners of female partners with HBV infection significantly higher than that for female partners without HBV infection (*p* < 0.05); no difference in male infertility for HBV infection vs no infection (45.2% vs 46.1%)
Cross-sectional (HIV) [110]	83 healthy men. Semen, blood	36/83 tested HIV positive; mean sperm motility reduced with HIV (34% vs 53%, *p* < 0.05); no significant difference in sperm count and sperm morphology between infected and noninfected men
Cross-sectional (CMV & HSV) [111]	83 infertile men Semen	CMV detected in 8 semen samples, HSV-II detected in four; no virus infection – induced specific morphological alteration were found; immature spermatogenic cells, with different manifestations of apoptosis were detected in all positive cases; Sperm concentration of positive group was significantly lower than the negative group (*p* < 0.5)
Case-control (CMV & HSV) [112]	808 infertile men. Semen	HSV more frequently found in infertile men’s whole ejaculate compared to controls (31% vs 17%, *p* = 0.049); HSV detection directly correlated with reduced amount of active motile spermatozoa (*p* = 0.0001) and smaller proportion of morphologically normal forms of germ cells (*p* = 0.002); CMV had no impact on motility and morphology of spermatozoids
Cross-sectional (HPV) [113]	117 infertile men. Semen	HPV did not affect DFI, sperm concentration, total sperm number, and total motility. Only progressive motility and morphology were found as significantly influenced by HPV; statistically significant difference in DFI between high-risk HPV (HR-HPV) and low-risk HPV (LR-HPV) genotypes
Cross-sectional (HPV) [114]	71 infertile men with HPV. Semen	Astheno-, asthenoterato-, oligoasthenotero-, oligoasteno-zoospermia detected in 56, 21, 16, and 6% cases respectively. HPV combination 6&11, 11&16, 16&33, 31&33 more often worsened several sperm parameters. HPV combination 6&11 significantly decreased progressive (6.20 ± 4.18%, 10.52 ± 5.66) and total (11.10 ± 5.95%,17.90 ± 6.92%) motility vs other HPV combinations. HPV combination 31&33 characterized by large decrease in total sperm count (12.78 ± 8.81 million, 21.82 ± 9.92 million) and sperm concentration (29.11 ± 21.54 million and 53, 35 ± 22.13 ppm) vs other HPV combinations
Cohort (HPV) [115]	117 infertile men. Semen	HPV prevalence 27.4%; No significant differences in sperm quality between HPV-positive and HPV-negative patients
Case–control (HPV) [116]	97 donors, 328 infertile. Semen and penile swab	High-risk HPV (hrHPV) genotypes detected in 28.9% of donors, 35.1% of IM. Penile swabs more frequently positive for hrHPV genotypes than semen samples in both IM (32.3% vs. 11.9%) and donors (26.8% vs. 6.2%,); hrHPV positive semen samples had lower median semen volume (2.5 vs 3 ml), sperm concentration (16 vs. 31 10^6^/ml) and total sperm count (46 vs 82 10^6^) than hrHPV negative. No association between penile hrHPV status and semen parameters

HPV *human papilloma virus*; HBV: *hepatitis B virus*; HCV *hepatitis C virus*; HIV *human immunodeficiency virus*; NS not significant; +ve positive; IL interleukin; STD sexually transmitted disease; IM infertile men; MDA malondialdehyde; CMV *cytomegalovirus*; HSV *herpes simplex virus*; DFI sperm DNA fragmentation

### Potential Pathophysiological mechanisms

Table 5 highlights the proposed individual pathophysiological mechanisms through which each sexually transmitted organism can affect male fertility as suggested by the included studies. Generally, STIs lead to the production of leukocytes and inflammatory mediators as well as reactive oxygen species, all these may affect semen parameters. Other mechanisms include direct suppression of spermatogenesis, scaring of seminal ducts leading to seminal duct obstruction, or alteration of sperm functions. Each organism can affect semen parameters through more than one mechanism.

**Table 5.** Proposed pathophysiological mechanisms of sexually transmitted organisms and male infertility.

Pathogen	Potential mechanism/s contributing to male infertility
**Bacterial**	
CT	Seminal deterioration due to generation of interleukin-1 (IL-1), leukocytospermia, ROS, OS and increased SDF. Immunological role also postulated with production of anti-sperm antibodies that affect different seminal parameters [117]; In Sertoli cells, *CT* might induce direct cell-damage, as evidenced by alteration of host-cell cytoskeleton, and, at the same time, remain within the cell for a long time, leading to a chronic infection [118]
NG	Mechanisms of male NG-mediated male infertility include spermatogenesis suppression, seminal tract obstruction due to accessory gland involvement, leukocytospermia and increase in OS [119, 120, 121].
Usp	Derangement in semen parameters [94, 122] due to disruptions of accessory gland functions, generation of OS and immunological reactions including, cross-reactions of antigens between human sperm membrane protein and Usp [123, 93,124]. Sperm DNA integrity was also reported to be significantly affected by Ureaplasma infection [89, 94]
TP	Syphilitic epididymitis can cause epididymal obstruction; syphilitic orchitis can damage seminiferous tubules; tertiary syphilis can cause testicular fibrosis; neurosyphilis can cause impairment in ejaculation/ erection mechanism [125].
MP	Semen parameters affected in infertile couples with Mycoplasma infection [126], due to the Mycoplasma effect on increase capacitation, acrosome reaction and sperm agglutination; mycoplasma bind to sperm membranes involved in sperm capacitation and acrosome reaction that is crucial in fertilization process [127]; binding hypothesized to impair physiological processes directly via binding or secondary to secretory products that may damage sperm membranes and affect overall sperm function [89]
NG, CT, MP	Can cause epididymitis which if not properly treated may lead to epididymal scaring and eventually obstructive azoospermia [128]; Infection extension to testicular tissue, even in asymptomatic cases, may affect spermatogenesis resulting in NOA [129].
**Viral**	
HIV	Spermatogenesis believed to be affected by presence of abnormal semen morphology and damage in DNA integrity; Cytokines and chemokines expression (IL-1, IL-4, IL6, IL-7, IL-8, GM-CSF, and MCP-1) cause inflammatory/ immunologic response believed to contribute [130, 131]
HSV	Semen parameters affected by HSV could be due to apoptotic effect on reproductive cells [111], decreased concentration of neutral α-glucosidase and citrate in infected semen samples [132], all leading to sperm motility reduction [112] and increase sperm abnormal morphology [133]
CMV	Male fertility believed to be due to gamete toxic effect, increase OS and leukocytospermia from inflammatory response on different semen parameters affecting sperm motility and morphology [134]
HBV	Believed to produce ROS which affects sperm function, apoptosis rate and membrane permeability leading to reduced sperm motility, vitality and ultimately infertility [135, 136]
HCV	Sperm aneuploidy, necrosis and ROS increase attributed to abnormal semen parameters in HCV infected patients; also reported to have hormonal imbalance role in infertility via affecting testosterone, estradiol, prolactin levels which bear on spermatogenesis [103, 137, 138]
TV	Theories of affected male fertility potential unclear, some believe due to cell membrane damage by perforin activity [139, 140], proinflammatory cytokine synthesis [141]; others reported cell destruction by phospholipase A2 secretion [142]

CT *Chlamydia trachomatis*; NG *Neisseria gonorrhea*; TP *Treponema pallidum*; Usp *Ureaplasma Species; HIV Human Immunodeficiency Virus*; HBV *Hepatitis B Virus*; HCV *Hepatitis C Virus*; HSV *Herpes Simplex Virus*; CMV *Cytomegalovirus*; MP *Mycoplasma*; TV *Trichomonas vaginalis*; STIs Sexually transmitted infections, OS oxidative stress; ROS Reactive Oxygen Species; SDF sperm DNA fragmentation; NOA non-obstructive azoospermia

## Discussion

Data on the effect of STIs on male infertility are heterogenous [40]. There is no recent systematic review that assessed a large number of eligible studies that collectively examined the full range of STI and their associations with male factor infertility [11]. This is despite the extreme importance of the topic to mens’ health, the notable focus on male infertility as a formidable challenge, and the increase in male infertility publications in recent years that could answer such a serious question. We undertook this task.

Our main findings were that the evidence seems equivocal. Using a quantitative assessment of the evidence, based on the number of studies, a roughly equal number of studies verified or refuted a significant association of each STI with detrimental changes in semen parameters. Hence, for each pathogen, in weighing the evidence for or against its association with male infertility, we further considered the level of evidence (LoE) of the given studies as to whether they comprised a lower (cross-sectional design – Level IV) or higher (case–control design – Level III) evidence level [143] (Table 6). Below we discuss each group of pathogens and their association with male infertility.

**Table 6.** Summary of the associations of 11 STIs and their supporting level of evidence*.

STI		Is STI significantly associated with deteriorated semen parameters?		Is prevalence of STI significantly higher among infertile than fertile men?		Summary			
		Yes	No	Yes	No	Yes, associated		No, not associated	
					LoE	Low	High	Low	High
C Trachomatis	Studies (n)	10	11	7	7	17		18	
	LoE	5 Level IV; 5 Level III	9 Level IV; 2 Level III	2 Level IV; 5 Level III	1 Level IV; 6 Level III	7	10	10	8
N Gonorrhea	Studies (n)	1	2	1	**–**	2		2	
	LoE	1 Level II	2 Level IV	1 Level III	**–**	0	2	2	0
Ureaplasma	Studies (n)	9	2	8	4	17		6	
	LoE	4 Level IV; 5 Level III	2 Level IV	1 Level IV; 7 Level III	4 Level III	5	12	2	4
Mycoplasma	Studies (n)	4	4	7	2	11		6	
	LoE	2 Level IV; 2 Level III	4 Level III	7 Level III	2 Level III	2	9	0	6
HPV	Studies (n)	3	2	1	**–**	4		2	
	LoE	2 Level IV; 1 Level III	2 Level IV	1 Level IV	**–**	3	1	2	0
HIV	Studies (n)	3	**–**	**–**	**–**	3		0	
	LoE	1 Level IV; 2 Level III	**–**	**–**	**–**	1	2	0	0
HSV	Studies (n)	2	2	1	**–**	3		1	
	LoE	1 Level IV; 1 Level III	2 Level IV	1 Level III	**–**	1	2	0	1
CMV	Studies (n)	2	1	**–**	**–**	2		1	
	LoE	2 Level IV	1 Level III	**–**	**–**	2	0	0	1
HBV	Studies (n)	1	1	1	1	2		2	
	LoE	1 Level III	1 Level III	1 Level III	1 Level IV	0	2	1	1
HCV	Studies (n)	1	1	**–**	**–**	1		1	
	LoE	1 Level III	1 Level III	**–**	**–**	0	1	0	1
T vaginalis	Studies (n)	2	1	**–**	**–**	2		1	
	LoE	1 Level IV; 1 Level III	1 Level III	**–**	**–**	1	1	0	1

LoE Level of evidence; Level IV evidence indicates cross-sectional studies; Level III evidence indicates case-controls studies; – review did not identify any studies that addressed this point; MS: Mycoplasma species; HPV: human papilloma virus; HIV: human immunodeficiency virus; HSV herpes simplex virus; HCV: hepatitis C virus; HBV: hepatitis B virus; CMV cytomegalovirus; *Clinical information access portal [143]

### Bacterial STIs

#### Chlamydia trachomatis

*CT* is the most common sexually transmitted organism in the male reproductive tract [144] with a global incidence of 2.7% in men and a prevalence ranging between 1.2% and 4% [145]. The partial acquired protective immunity to *CT* makes the affected patient prone to recurrent infections [146,147]. Studies report conflicting results of the associations of *CT* with semen parameters. This inconsistency was found in the reported percentage of *CT* detected in semen samples of infertile couples compared to fertile controls. Table 2 shows that while some authors found a statistically higher incidence of detection in infertile men, others reported no difference between both groups. Similarly, the association of *CT* with deterioration of semen analysis results showed much discrepancy between studies (Table 2). Such inconsistencies are probably due to the variations in organism detection methods and time between infection and analysis. Collectively, based on an equal number of studies with near similar levels of evidence that assessed semen parameters and differences in prevalence, the findings of Table 6 confirm that the association of CT with male infertility remains equivocal in verifying or refuting an association of CT with male infertility.

#### Neisseria gonorrhea

*NG* is one of the most prevalent STI, with almost 106 million new diagnosed cases per year as reported by the WHO in 2012 [88,144]. Very sparse data exist on its association with male infertility. The current systematic review found only four studies on the effect of *NG* in infertile couples. One prospective cohort study on 45 gonococcal epididymo-orchitis infected men found that bacterial gonadal infection may result in permanent azoospermia or oligozoospermia, and that 60% of the cohort two years after infection showed abnormal semen parameters (LoE II) [65,90]. Another case–control study reported a significant presence of *NG* (6.5%) in infertile group compared to its absence in semen of fertile men (LoE III) [50]. On the other hand, two cross-sectional studies did not detect semen parameters’ differences among infertile men with and without *NG* infection (LoE IV) (Tables 3 & 6). Due to the limited number of studies and their level of evidence, the current review is unable to confirm an association between *NG* and male infertility or otherwise.

#### Ureaplasma Species

Ureaplasma species are considered normal flora inhabiting the male and female body [148]. In controlled count, they are asymptomatic and exist in balance without causing problems, but when their numbers increase, they can cause urogenital tract infection that affects semen [122]. Prevalence of Ureaplasma in infertile couples ranges between 5% and 42% [148]. Its role in male infertility remains contradictory, with studies suggesting no effect on or derangement of semen parameters (Table 3). The present review identified 21 studies on Ureaplasma and male infertility. The findings confirm an association of Ureaplasma with male infertility, based on a larger number of studies with higher levels of evidence reporting higher prevalence of Ureaplasma in infertile men and deteriorated semen parameters in Ureaplasma-infected men (Table 6).

#### Mycoplasma

Several studies found significant prevalence of Mycoplasma species especially *Mycoplasma hominis* in infertile couples, although it was asymptomatic. The differences in prevalence was attributed to difference in detection methods utilized [149]. Treating the infection showed significant improvement in a case–control study, where 58.3% pregnancy rate was achieved in infected couples after four months of treatment with improvement of semen parameters, ROS production and total antioxidant capacity to the normal ranges among these couples [89]. Various studies reported contradictory findings (Table 3). The current review seems to suggest an association of Mycoplasma with male infertility, based on a larger number of studies with higher levels of evidence reporting higher prevalence of Mycoplasma among infertile men. Notwithstanding, the evidence on deteriorated semen parameters among Mycoplasma-infected men was equivocal (Table 6). However, taken together, the summary of the evidence appears to be more inclined towards a positive association.

#### Treponema pallidum

About 12 million new cases of syphilis are reported per year worldwide [150]. Despite that the association of *Treponema pallidum* with male infertility is a matter of debate, yet many theories support the strong effect it has on infertility [149,151]. However, the current systematic review could not identify any original study that set out to assess the association of syphilis with male infertility.

### Viral Infections of Semen

#### Human Papilloma Virus (HPV)

HPV is one of the most common viral STIs affecting men and women [152,153]. Numerous strains are linked to the infection of genital tract [154]. The effect of HPV on women is different from men, where mostly the virus is eradicated and in a small percent, it can be isolated in semen and other male reproductive organs [155]. Controversy exists about HPV and fertility. Preserved fertility was reported by Lee *et al*. [13] and Schillaci *et al*. [156] with no effect of semen parameters, while others [100] found significantly reduced sperm motility in HPV-positive men, proposing that antisperm antibodies and lower fertilization capacity rates after HPV infection could explain the effect on male infertility. Another study of 1,003 patients reported that 28.6% men were positive for HPV in one or more genital samples [67], with significantly reduced motility and increased abnormal sperm morphology in the HPV co-infection individuals [67]. However, due to the small number of studies, their low level of evidence and the lack of fertile controls, the effect of HPV on male infertility is still under debate (Table 6).

#### Human Immunodeficiency Virus (HIV)

Semen is the main vector in HIV transmission in men. The effect of HIV on fertility depends on viral load, immunity status of the infected individual, stage of disease, and being on treatment or otherwise [149]. A case–control study of 250 HIV-positive men and 38 fertile controls reported significant reduction in median sperm concentration and median sperm motility [105]. Another two cross-sectional studies observed that semen parameters were significantly correlated with different CD4+ cell numbers, and overall sperm motility and vitality were lower in patients with CD4+ <350/ml, and reported that HIV caused hypogonadism, which affects spermatogenesis and sperm count [93,157] (Table 4). The insufficient number of studies that the current review identified mitigates against drawing a solid conclusion for the relationship between HIV and male fertility (Table 6).

#### Herpes Simplex Virus (HSV)

HSV-2 commonly causes genital herpes. It has been isolated from semen, testicular and prostatic tissue of infected men [158,159]. Fluctuation of HSV prevalence is attributed to the different detection modalities, reporting HSV DNA in 49.5% of infected men’s semen by nested PCR [150], 3.7% detection with semi quantitative PCR [132], and 25% detection with rapid culture method [160]. The current systematic review found four studies on the HSV-infertility relationship (Table 4). The insufficient number of studies that the current review identified renders deriving a valid conclusion for the relationship between HSV and male fertility difficult (Table 6).

#### Human cytomegalovirus (HCMV)

Immunocompromised and transplant patients are most affected by HCMV. The virus can infect male reproductive organs and can be isolated from semen together with other body secretions (e.g. urine, feces, vaginal/cervical secretions, blood and milk) [161–163]. A laboratory study of the effect of HCMV on ejaculated sperm reported no effect on sperm motility [164], and another case–control study suggested no relationship between HCMV infection and fertility potential [112]. Conversely, a study of 83 semen samples of infertile men found significant effect of HCMV on sperm concentration [111]. Others [107] reported a prevalence of 17.7% HCMV in 232 semen samples of men attending the infertility clinic with chronic inflammatory urogenital infections. A reduction in sperm count compared to other groups of different organisms combined was observed [134] (Table 4). We are unable to conclude the relationship between HCMV and male fertility due to the scarce number of studies that the present review identified (Table 6).

#### Hepatitis-B Virus (HBV)

Controversies about HBV and male infertility have been discussed in the past several years. Table 4 shows that in a small cohort of 15 HBV infected men there was no difference in sperm parameters between patients and normal controls, with only an increase of sperm necrosis in the infected men [106]. Another retrospective study among 831 infertile couples detected HBV in 7.3% of men, but no statistically significant difference between infected and non-infected couples [109]. On the other hand, others observed a significant reduction in semen parameters with HBV, and a significant increase of IL-17, IL-18, and malondialdehyde levels in subjects with HBV compared to normal males [104]. Another retrospective study of 5138 men with HBV noted a 10-year increase in the infertility incidence among this cohort [108]. Based on the present review, it is not prudent to conclude any relationship between HBV and male fertility due to the very small number of studies with near levels of evidence (Table 6).

#### Hepatitis C Virus (HCV)

Despite a prevalence of HCV in seminal fluid of up to 30%, the rates of sexual and vertical transmission were low at 5% [165]. Research compared semen samples of 57 HCV infected men to 40 fertile controls and found that mean sperm count, and mean sperm motility were significantly reduced, and mean sperm abnormal forms significantly increased with HCV [103]. On the other hand, a study of 3 HCV infected men found no difference, in sperm parameters between patients and normal controls, with only an increase of sperm necrosis in the infected men [106] (Table 4). As there were only these two case–control studies, any relationship between any relationship between HCV and male fertility remains to uncovered (Table 6).

#### Protozoal Infections (Trichomonas vaginalis)

Trichomonas vaginalis affects men and women. Symptoms vary by gender, with men mostly asymptomatic because of the cytotoxic prostatic zinc which counteracts the organism pathogenicity [142]. The effect of trichomoniasis on male fertility remains controversial. In vitro studies [83] reported no effects on sperm motility, while others [84] reported opposite results. A study also found significant reduction in sperm motility and normal morphology in trichomoniasis patients and noted significant improvement of these parameters with a single course treatment in 25 cases [85]. Several theories upon which *T. vaginalis* affects male fertility potential have been postulated [139–142] (Table 3). Given that there were only three studies that the current review identified, any relationship between *T. vaginalis* and male fertility is still to be discovered (Table 6).

The current review has limitations. It was limited to studies published in English language. The review was unable to precisely differentiate the associations of STI on male fertility during the active infection stage vs the long-term consequences of STI on male fertility, as most of the included studies did not clearly specify the duration between the start of STI and the semen analysis. The review has also many strengths. It bridged the identified knowledge deficits to provide a state-of-the-art comprehensive in-depth assessment of the association between STIs and male factor infertility employing the largest number of original studies to date and grading the evidence not only based on number of studies only but also on the actual level of evidence of each study.

## Summary

Taking the current review’s finding pragmatically, in answering the first question ‘Are STIs significantly associated with detrimental changes in semen parameters?’, the evidence seems equivocal. The nearly equal number of studies that examined each of 11 sexually transmitted pathogens, and their near-equal levels of evidence, preclude against any solid confirmation or refutation of a significant association of the given pathogens with detrimental changes in semen parameters. The only exception was for Ureaplasma, where the number of studies and their levels of evidence supported an association with male infertility.

In answering the second question ‘Is the prevalence of STI significantly higher among infertile compared to fertile men?’, there was insufficient evidence to support or deny a significant association of a difference prevalence of the given STI being significantly higher among infertile than fertile men. There were two exceptions: for Ureaplasma and Mycoplasma, the number of studies and their evidence levels demonstrated support in favour of an association with male infertility. In considering both questions together, the evidence appears to be equivocal for most STI, except for Ureaplasma and Mycoplasma, both of which could affect infertility in men.

On the other hand, looking holistically at the state of the science on the association between STIs and male infertility, the evidence base leaves much to be desired. Whilst the topic is extremely important and intuitional, several points require attention. First, in terms of number of studies, we identified only 70 articles over 53 years. Second, as regards to the spread of articles over pathogens, the 70 articles report on 11 pathogens, with 48 of these articles focusing on 3 pathogens. Third, as for study design, the search did not identify a single prospective long-term study, with most studies being level III or IV level of evidence. The exceptions we Ureaplasma and Mycoplasma, where the evidence convincingly suggests their associations with infertility in men.

The current review revealed several interesting findings: very few studies examined the short- and long-term associations between fertility and very common organisms such as *Neisseria gonorrhea* or viral STIs. Likewise, no studies were identified that appraised the relationships between Treponema pallidum and fertility.

## Conclusions

Inconsistencies exist as to the role of individual STIs in male infertility. The present systematic review carefully analyzed the identified studies which enabled a better understanding of the various mechanisms by which sexually transmitted pathogens can affect male fertility. The current state-of-the-art evidence suggests the positive roles of Ureaplasma and Mycoplasma in the pathogenesis of male infertility. This is substantiated by the large number of studies with the appropriate level of evidence. For the remaining STIs, there is currently insufficient solid evidence, to support whether they explicitly impact male fertility. Future clinical and in-depth mechanistic inquiries should use study designs of high level of evidence and sufficient sample sizes are required to enhance the evidence base for these STIs and their relationships to infertility.
